# A review of the physiological and psychological health and wellbeing of naval service personnel and the modalities used for monitoring

**DOI:** 10.1186/s40779-016-0112-3

**Published:** 2017-01-18

**Authors:** Cliodhna Sargent, Cormac Gebruers, Jim O’Mahony

**Affiliations:** 1 Cork Institute of Technology, Rossa Avenue, Bishopstown, Cork Ireland; 2 National Maritime College of Ireland, Ringaskiddy, Cork Ireland

**Keywords:** Navy, Military, Seafarer, Psychological-physical fitness, Fatigue, Nutrition, Substance abuse

## Abstract

Naval cohorts rely heavily on personnel to ensure the efficient running of naval organisations. As such, the wellbeing of personnel is essential. In an occupational setting, naval service personnel experience a variety of physiological and psychological stressors. Most naval services arrange annual physical fitness and body composition tests to ensure the physical readiness of personnel. However, these tests only evaluate a small amount of physiological capabilities. Components such as aerobic and strength capabilities are assessed, however, other components of physical fitness such as speed, agility, anaerobic capacity and flexibility are not. In addition to the physical capabilities, personnel are impacted by fatigue, nutrition and psychological stressors such as copping in stressful situations or dealing with time away from family and friends. This review will discuss the physiological and psychological factors that affect personnel’s wellbeing. In addition to this, it will also evaluate the methods that are used to assess both physiological and psychological wellbeing.

## Background

For many seafaring nations, the Naval Service is an important part of the military cohort. As a result, nations use several resources to ensure that naval services operate efficiently. Although this operational efficiency includes major resources, such as warships, the functionality of every naval cohort heavily relies on its personnel. Both financial and time resources are used to occupationally train personnel. Therefore, the wellbeing of Naval Service personnel is essential.

Research has shown that being at sea can have a large impact on the physical and mental wellbeing of seafarers, which includes naval service personnel. Although an individual’s physical and psychological capabilities impact their wellbeing, there are many additional factors that can affect health.

First, obesity has become a global issue. Military cohorts are generally expected to be fit and have a good body composition, however; it is unclear whether this is true among naval cohorts who contend with different working conditions compared to their military counterparts. The World Health Organization (WHO) established body mass index (BMI) guidelines [1] that are useful for the general population. However, if naval service populations have muscular body compositions, then it is important to consider alternative methods of body composition analysis.

Physical fitness is another variable that may affect wellbeing at sea. There are many different components to physical fitness, but in an occupational setting, it is important to examine how physical fitness affects occupational capabilities. Within the naval service, tasks such as on-board firefighting and casualty carry have occupational relevance [2]. However, there is a need for additional research to assess the physiological stressors that are placed on individuals in certain occupations in the naval service.

There is also a psychological aspect to the occupational stressors that naval service personnel experience. Many researchers have focused on stressors that are caused by deployment into war zones. However, the overall impact of additional psychological stressors should be assessed within naval service populations. Questionnaires are widely used to assess psychological wellbeing, but is unclear as to which questionnaire is the most beneficial in a naval service cohort.

Most research on military organizations does not separate different military cohorts into the navy, army and air force. However, this type of research could provide valuable insight into how these cohorts differ and why they should be treated as separate entities. The aims of this review are 1) to analyze the existing research on naval service personnel and 2) to provide a better understanding of the lifestyle and health factors that impact life at sea. In addition to data on naval service populations, this review will compare outcomes among other military cohorts. When there is a lack of data on naval service populations, we will include research on general seafarers to provide a better understanding of the demands of being at sea. Currently, there is no single document that has reviewed the research on all three cohorts or the major factors that impact their health and working conditions.

## Body composition

Obesity and being overweight can be defined as abnormal or excessive fat accumulation that may impair health [1]. In many parts of the world, obesity and being overweight have become major health problems, with rising rates in many countries [1, 3, 4]. In 2011, 23.4% of the adult Irish population were classified as obese [5]. Obesity is especially prevalent in certain occupations. In the seafaring industry, several factors contribute to obesity, including an increasingly sedentary lifestyle, easy access to high quantities of food [4] and a lack of control over the quality of food that is served [6]. Being overweight may reduce the ability to perform daily duties, and being on a ship adds an extra danger due to the potential for reduced mobility in an emergency situation [7]. Consequences of being overweight or obese include a risk for poor mental health, cardiovascular disease, musculoskeletal disorders, some cancers, stroke and diabetes [1, 4]. There is an increased risk for these health problems if they occur when the individual is on-board a ship, as there is limited access to professional medical help [7]. A study in the Netherlands found that 52% of those who were declared unfit in the seafarer’s medical qualification had cardiovascular conditions and morbid obesity [8]. This research shows that being overweight can affect one’s ability to effectively perform their job, and may prevent continued employment. This loss of employment will financially affect the employer and results in a loss of experience.

BMI is one of the most commonly used methods for identifying adults who are overweight or obese as it is easy to assess and incurs minimal costs [1, 3]. It is calculated using a person’s weight in kilograms (kg), which is divided by the square of a person’s height in meters (kg/m^2^) [1]. In many older studies, BMI is divided into four different categories: a BMI of less than twenty is below normal weight, a BMI of greater than or equal to twenty and less than twenty five is normal weight, a BMI of greater than or equal to 25 kg/m^2^ and less than 30 kg/m^2^ is moderately overweight and a BMI of greater than or equal to 30 kg/m^2^ is obese [7]. However, in 2006, the WHO created a fact sheet on obesity and being overweight, which suggested that there should only be three BMI categories: underweight and normal weight have a BMI of below 25 kg/m^2^, overweight has a BMI of 25–29.9 kg/m^2^ and obesity has a BMI of 30 kg/m^2^ or more [1].

An unpublished undergraduate study that examined being overweight and obese in the Irish Naval Service found that 48.6% of 820 cases were overweight and 16% were obese [9]. This figure was 5.5% above the national average that was outlined in the 1990 Irish National Nutrition survey. This study is consistent with research on Danish seafarers, which found a higher percentage of seafarers were overweight or obese compared to the general population in the same category [7]. In this study, 64% of male seafarers were overweight and 19% of all participants were obese [7]. A similar study on Danish seafarers found that 70.5% of male seafarers were overweight, which may indicate that rates of being overweight are increasing [4]. This problem was also found in a study on U.S. service personnel, in which the number of personnel who were in the overweight and obese categories increased between the years 1995 and 2005 [10]. However, an additional study that was conducted by Bray et al. [11] found that the percentage of those in the overweight and obese categories had not further increased in 2008, and there was a decrease in the percentage of those who were overweight among those who were under twenty years old. This study also found that the percentage of personnel in the overweight category was higher in the navy and coast guard (both 63%) than in the marine corps (55%) or the air force (59%) [11].

There may be a higher percentage of overweight individuals in the navy due to several factors. For example, in the U.S. Navy, after a period of deployment, there was an increase in the percentage of males who exceeded the weight recommendations, which further increased after a second deployment [12]. In line with this finding, a study on the U.S. military found that there was an increased risk of clinically important weight gain among those who had less education, were overweight at baseline, and had experienced deployment with combat exposure or were in active duty [13]. Therefore, it is possible that the on-board ship period may result in high levels of being overweight among the navy and coast guard.

Research that investigated the Irish Naval Service also found that, in the 18–35 age category, 58.4% were either overweight or obese, in the 36–50 age category, 78% were overweight or obese, and in the 51–60 age category, 95.6% were overweight or obese [9]. As a result, as age increases, so does the number of overweight or obese individuals. Similarly, a study on the U.S. Navy found that BMI increased with age in both genders, however, this increase was more pronounced for males. A study on U.S. service personnel over a 10 year period found that the number of individuals who were clinically diagnosed as overweight increased in all age groups [14]. A separate study on U.S. service personnel found that the percentage of those who were classified as overweight in the under twenty age group increased between 1995 and 2005 from 28 to 45%, but decreased to 35% in 2008. However, between 1995 and 2005 the percentage of individuals over 20 years old who were classified as overweight increased by 11%, but, by 2008, this level remained the same [11]. Although this data may indicate that the rates of obesity and being overweight are stabilizing in people who are over 20-years-old, being overweight or obese can still affect a person’s ability to perform occupational tasks. A study that examined the relationship between age and BMI on seafarer’s work found that there was a larger effect of a high BMI on work ability in older individuals [15]. This study indicates that being overweight or obese effects older individuals more than younger individuals.

In 2008, a study on males in the U.S. Navy found that 69% of those assigned to small submarines were overweight or obese, 66% of those assigned to large submarines were overweight or obese and 63% of those assigned to aircraft carriers were overweight or obese [16]. These figures indicate that the more confined the vessel, the higher the level of obesity or being overweight. This study also found that the mean BMI for each group was similar to the general U.S. population. However, a national health and nutrition examination survey showed that 27.5% of those surveyed in the U.S. general population had a BMI that was greater than 30 kg/m^2^ [17], which is much higher than the rates of 18% among small submarine, 17% of large submarine and 15% of aircraft carrier personnel [16]. A study on the Irish Naval Service classified individuals into five different occupational categories to assess levels of being overweight, which included fleet, shore command, officer command, the naval college and naval headquarters. The fleet had the highest number of obese and overweight cases, as 43% were overweight and 50% were obese [9]. This study did not provide information about the length of time that these individuals had been working in the fleet or their previous work assignments. This information would be critical for establishing the causes of high levels of being overweight and obese.

Although being overweight in the armed forces or at sea in general can have an effect on an individual’s ability to perform required occupational activities, treatment for being overweight or obese can also lead to high healthcare costs. Research found that the inpatient care costs for the U.S. Navy was estimated to be $5,842,627 for the top 10 obesity-related diagnosis groups [18]. Research in 2007 estimated that spending on medical care associated with weight and obesity for the entire U.S. department of defense was in excess of $1.1 billion [19]. To combat these rising costs, research has investigated methods of prevention and found that a multi-component approach to obesity in the military is cost effective [20]. This type of research has included implementing intervention programs in several populations. These programs include LEAN [21–24], lifestyle change, individual readiness, fitness excellence and eating healthy (LIFE) [25] and fat loss and exercise (FLEX) [26]. Research that evaluated these programs found that they were developed for a U.S. population and may not be suitable for other military populations [20].

### Measuring body composition

Several military cohorts use BMI as a measure of body composition [27]. In the U.K. Armed Forces, an acceptable range for BMI is from 18 to 28 kg/m^2^, while the maximum can be 30 kg/m^2^ for females and 32 kg/m^2^ for males. Those who are under eighteen can have a maximum BMI of 27 kg/m^2^. For entry into the Australian Defense Forces, a BMI of 18.5–30 kg/m^2^ is ideal, while the maximum entry allowance is 32.9 kg/m^2^ [28]. However, it does recognize the WHO standards and believes that anyone with a BMI of 25 or over is overweight [28]. In the Irish Defence Forces, BMI is required to be calculated during the Annual Health Assessment [28]. In comparison to the levels outlined by the Australian Defence Forces, the Australian Maritime Safety Authority states that if a person has a BMI of over 30 kg/m^2^, he or she will be required to demonstrate an ability to climb ladders, go through hatches and not exceed the weight safety limits for the rescue equipment [29]. Rather than using BMI, the U.S. Navy assesses whether an individual is ‘within standards’ or ‘out of standards’ during a physical fitness assessment by combining the maximum weight for height and the navy circumference measure, which includes the neck and abdominal circumference for males and neck waist and hips for females [30]. Studies have shown that the maximum measurements allowed according to U.S. standards correspond to a BMI of between 25.9 and 29.9 kg/m2 [31–33]. However, a study comparing BMI to the current method that the U.S. Navy uses found that 296 of 3,306 people were classified as within acceptable limits, despite epidemiological evidence that the individuals were at a significantly higher risk of morbidity and death resulting from chronic diseases [30].

However, although BMI has some advantages, it has been criticized for its lack of validity in certain populations. Studies have shown that an individual with a high muscle mass can have a high BMI even though there is a low percentage of body fat [34, 35]. As a result, when using BMI to evaluate the body composition of trained individuals, it may be necessary to use additional measures [36]. In many seafaring occupations, when a seafarer meets a measured level of obesity, additional assessments are administered. In general seafarers, additional measures include ergometric tests (measures of work or energy) or blood tests that assess cholesterol levels, fasting glucose and triglycerides, which are indicators of coronary risk [37]. In the Irish Naval Service, skinfold measurements can confirm BMI results [27]. In the U.K. armed forces, when a certain level of BMI is obtained, additional tests include waist circumference and fitness. This action is similar to the Australian Defence Force, in which waist circumference is used as an additional indicator of the risk for morbidity [28].

There are several different methods for measuring a person’s body composition, however; research has identified three different validity methods [38]. The most accurate method is direct measurement, however; this method involves dissecting the subject and is not feasible in a living population. The second method involves indirect measurements, which are usually performed in a laboratory setting and include densitometry, computed tomography (CT), magnetic resonance imaging (MRI) and dual energy x-ray absorptiometry (DXA) exams. These methods have high accuracy levels. However, the required equipment is expensive, importable and requires a high level of technical skill. These factors may make these methods difficult to use in studies that include large samples. The DXA is one of the most commonly used method for calculating body composition. The DXA measures differences in absorption at two low x-ray energies to estimate bone mineral content and soft tissue composition [39]. This method, along with air displacement, underwater weighing, and labelled water techniques have been used as reference methods because they are highly accurate [40–43].

The third validation method for body composition consists of double indirect methods, which include anthropometry and bioelectrical impedance analysis (BIA) [38]. BIA measures how the body conducts electricity, and is based on the assumption that fat-free mass is a good conductor and fat mass is a poor conductor of electricity. This method is inexpensive and easy to use, however; several factors can affect the accuracy of the results, such as consuming large amounts of water prior to testing. There has also been little research on the scales or hand held devices that are used to assess BIA. Anthropometry is the study of the size, shape and strength of the human body, including mass, volumes, mobility, proportions, centers of gravity and the inertial properties of the entire body and body segments. Anthropometry uses methods, such as waist circumference, waist-to-hip ratio and skinfold measurements [44, 45]. These methods are most often used in large sample sizes when there is a need for a quick measure or when no financial resources are available [45].

Although abdominal obesity alone is not an indicator of body composition or health, a study on metabolic syndromes in the Brazilian Navy found that 35% of subjects had abdominal obesity and that it was a marker of a dysmetabolic state as well as a partial cause of metabolic syndrome [46]. The waist circumference must be less than 80 cm in females and 92 cm in males. In comparison, the Australian Defence Forces have the same waist circumference cut off levels for females but set the cut off levels for males as 88 cm [28].

Skinfold measurements have been widely used because they are quick, inexpensive and require limited training and standardized procedures for obtaining reliable results [39]. This type of measurement uses calipers to measure subcutaneous fat in specific body sites [39]. These measures indicate the total percentage of body fat because subcutaneous fat represents 40–60% of total body fat [39]. Using standardized methods and selecting the appropriate equations based on the specific population being tested increases the accuracy of this measure [47, 48]. However, there are also limitations in using skinfold measurements. Research found that the accuracy of assessing the percentage of body fat at the individual level using skinfold measurements was poor compared to the results from a DXA scan [39]. However, skinfold thickness measurements are better predictors of the percentage of body fat than other anthropometric variables, such as BMI [49].

To increase the accuracy of skinfold measurements, data must be entered into the most appropriate equation for a particular population. Several studies have used skinfold measurements to ascertain body composition in military populations [50–53]. In these studies, equations include the Durnin and Womersley equation [52, 54].

However, the most common equation is the Jackson and Pollock equation [55, 56], which has been used in both male [51, 53, 57] and female military populations [50, 53]. As a result, when skinfold measurements are conducted in a mixed military population, the Jackson and Pollock equations are the most suitable. Although skinfold tests are not consistently used to measure body composition in the Irish Naval Service, the Irish Defence Forces claims that they can be used as needed [27].

## Physical fitness

Although physical activity has both physical and mental benefits, there has been a decrease in physical activity in both low-medium and high income countries [58, 59]. This decrease has been attributed to several factors, including a lack of leisure time, the stress of daily living, the introduction of new technology, and changes in work processes [59, 60]. Although some of the occupational activities at sea still have elements of hard physical labor, as with many occupations over the last decade, there has been a decrease in the energy expenditure among seafarers [61].

Although occupational physical activity has been negatively associated with absences from work, leisure time physical activity has been associated with fewer absences from long term sickness [62]. A study on the Brazilian military found that higher levels of job stress were associated with high levels of occupational physical activity, but not high levels of leisure time activity [63, 64]. These studies indicate that the benefits of physical activity occur as a result of participating in leisure time activity and not occupational physical activity. Therefore, increased leisure time activity could positively affect employees in the workplace. Additionally, studies have found that the inability to exercise is one of the main stressors associated with working at sea [65].

It has also been suggested that increasing exercise, along with reducing smoking and alcohol consumption, may promote seafarers’ health and reduce the risk for stress related diseases [65]. A study on Icelandic seafarers found that providing assistance with commencing and maintaining a more physically active lifestyle, along with providing on-board fitness equipment, led to a significant reduction in fishermen’s potential health risks [66]. Research on general populations has ascertained the effects of fitness training and found that these types of intervention programs can be cost effective [67, 68]. For some occupations, such as firefighters and the police, introducing a health and fitness program can lead to a reduction in the time needed to perform occupational activities [69, 70]. Among military personnel, these programs have led to an increase in performance during physical fitness testing [71]. Positive cost effective outcomes have also been evident in military settings. For example, implementing two different training programs for those who failed the army physical fitness test enabled personnel retention [72]. Because recruiting and training military personnel can be very costly and time consuming, it is important to retain personnel [72]. This type of training can prevent costs related to retention, while increases in physical fitness can prevent the risk of injury or illness [73]. For example, a study on the Norwegian Navy found that physical activity is associated with a low prevalence of musculoskeletal disorders [74], which is a common disorder in military settings [75]. These studies indicate that when an employer allows employees to perform leisure time activities, there are long-term financial benefits for the employer. However, physical training can also cause injuries when programs are not correctly implemented or do not have leadership support and understanding [73].

Research has suggested that leaner personnel use less physical working capacity for occupational activities than personnel classified as overweight [76]. In addition, physical testing failure is associated with individuals who are obese or overweight [77]. Additionally, smokers and individuals who have a higher BMI are associated with slower 1.5 mile run/times, as well as poorer upper strength and core fitness [78]. A separate study found that individuals who had a higher body fat level performed better on the push-up test but worse on every other test compared to individuals who have low body fat levels [79]. These studies indicate that those who have a body fat that is below the overweight level can physically perform better on occupational tasks. As a result, many occupations that require physical occupational activities also conduct physical fitness assessments.

In the general seafaring population, a physical fitness test is only conducted when the individual has conditions that include a high or significantly low body mass, severely reduced muscle mass, musculoskeletal disease, pain or limitations in movement, a condition following an injury or surgery, lung disease, heart and blood disease or some neurological diseases [80]. Research has found that when exposed to stressful stimuli, men who have better cardio respiratory fitness respond more calmly [81]. This data indicates that employers could justify implementing a physical fitness test in stressful occupations to assess coping ability.

Although physical fitness testing is not mandatory in the general seafaring population, in many navy populations all personnel are required to perform an annual mandatory physical fitness test [82]. Physical fitness is defined as a set of attributes that individuals have or can achieve, which is related to their ability to perform physical activity [83]. Physical fitness is often measured through assessing aerobic capacity, muscular strength and muscular endurance [84]. The Irish Naval Service physical fitness testing generally consist of two parts. The first part is a test of body composition. In the second part, personnel are required to complete a one-minute push-up test and a one-minute sit-up test to test local muscular endurance and a 2.4 km run to test cardiovascular endurance. This form of testing is also used in the U.S. Navy, however; there are alternatives to the 1.5 km run as a test of cardio vascular endurance in the form of a swim, an elliptical trainer or stationary bike [85]. Similarly, the British Navy provide alternatives in the form of the Multi Stage fitness test (MSFT) and the Rockport walking test (this test is only used for older age groups or those who have medical conditions that prevent maximal exercise) [86]. A separate strength test is also used in the British Navy, which involves carrying two 20 kg weights over a distance of four 15 m shuttles [86]. This test has seeks to simulate the aqueous film forming foam drum carry that is required during fire-fighting tasks on-board vessels [86].

When physical fitness testing standards are continuously not achieved, then the military organization may be forced to act. This action could involve consequences, such as unfavorable administrative actions, preventing promotions or involuntary separation and discharge [72]. However, some researchers believe that these tests are biased against heavier personnel who have a greater body mass [87–90]. This bias is related to the current tests that use body weight as the resistance, thus, the individual who is being tested is always carrying their own body weight. The issues occur when an individual who is 75 kg scores better on the physical fitness tests than one who weighs 100 kg, and, as a result, the 75 kg individual would get the promotion. However, the 100 kg individual may be able to rescue a heavier casualty than the 75 kg in an occupational setting, but the tests do not account for this capacity [91]. This research indicates that these tests may be inappropriate for assessing an individual’s work ability if load carrying is part of the occupational activities [89]. However, having a higher body mass is different from being ‘over-fat,’ in which the ability for load carriage is reduced among military personnel [92].

### Physical fitness testing

The Irish Naval Service tests two different physical aspects of physical fitness: aerobic capacity and muscular endurance. However, there are many different physical elements that can be analyzed during physical testing, including an individual’s flexibility, power, agility, muscle endurance, strength, speed, anaerobic conditioning and aerobic conditioning. When many aspects of physical fitness are assessed, it becomes easier to ascertain if changes in a specific area have occurred over a given period of time. However, it has been recommended that the total number of all physical fitness tests conducted within a single battery (e.g., tests of flexibility, speed, muscle strength, power, coordination) should not exceed 10–12 due to the limited time available and possible problems with participant motivation [93].

#### Flexibility

Flexibility can be defined as the ability of a muscle to lengthen and allow one or more joints to move through a range of motion [94]. The benefits of enhanced flexibility can include improved athletic performance [95, 96], a reduced risk of injury [97] and pain relief [98]. Flexibility can be affected by several factors, including a warm up that enhances muscle elasticity facilitating flexibility, or vibratory stimulation, which encourages the muscle to relax [99]. There are also several ways to measure an individual’s physical flexibility depending on which part or parts of the body are being assessed. Many flexibility tests examine the trunk and the lower part of the body because maintaining hamstring and lower back flexibility may prevent lower back problems, postural deviations, gait limitations, the risk of falling and acute or chronic musculoskeletal injuries [100]. Similarly, during basic training in the U.S. Military, research found that including exercises to increase hamstring flexibility could reduce the occurrence of overuse injuries in the lower extremities [97]. This reduction in the occurrence of injuries could indicate that including flexibility training in the everyday life of military personnel may reduce the risk of musculoskeletal injuries, which have high financial and personnel costs. However, a study on the U.S. Army found that an increased risk of injury is related to low and high levels of flexibility [101]. Thus, it is the extremes in flexibility that lead to an increased risk of injury, and injury prevention programs should target both extremes.

Laboratory and field settings can be used to test for flexibility in the lower extremities. Laboratory settings are often highly accurate. In this setting, flexibility is measured using devices, such as electrogoniometers, flexometers, inclinometers and 3D systems for kinetic analysis, which directly measure a certain aspect of an individual’s flexibility [102–105]. However, these tests are complex, expensive and can be time consuming with larger groups. Therefore, there are alternative field tests for the lower extremities that measure flexibility and are simple and inexpensive [106]. Field tests include sit and reach, leg raises in a supine position, sideways leg splits, single legged knee bends and lengthwise leg splits [106]. However, there is a lack of data on the validity and reliability of these tests, and each test may measure a different aspect of flexibility. The sit and reach test measures flexibility in the hamstring and lower back muscles, leg raises in a supine position measure flexibility in the hamstring muscles, sideways leg splits measure flexibility in the adductor muscles, single knee bends measure flexibility in the hip flexor muscles, hip abduction tests assesses flexibility in the adductor muscles and lengthwise leg splits measure flexibility in the hamstring and quadriceps muscles [106]. Although each of these tests uses different measures, one study found that they are sufficiently valid and reliable for replacing laboratory measurements [106].

The sit and reach test is one of the most commonly used flexibility tests. There are many versions of and protocols for the sit and reach test that have been used with several populations. These versions include the original sit and reach, chair sit and reach and the back saver sit and reach. Each of these tests has moderate validity for hamstring extensibility and poor validity for lower back flexibility [107]. In a comparison between the back saver sit and reach and the original sit and reach, research suggested that the original sit and reach had better concurrent validity and easy-to-use protocols [108]. In addition, when only one test of flexibility is being measured, the sit and reach test may be the most suitable. The results from this test are affected by the flexibility of one joint as well as several other factors, which include the lower back, hamstring and shoulder flexibility as well as longitudinal body dimensions [107], which indicate overall flexibility.

#### Power

The movement of jumping requires complex motor coordination between the lower and upper body segments. The propulsive action of the lower limbs while performing a jump has been used to evaluate the explosive characteristics of elite athletes and sedentary individuals as well as their ability to generate power [109]. Because it is important for assessing lower limb biomechanical properties, sports experts typically use valid laboratory-based instruments, such as photoelectric cells [110–112], force platforms [113–116] and contact mats [117] to gather information about jumps. These instruments may not be suitable for repeated use with large groups because the equipment is expensive, however; they are frequently used in the field by physiologists. The instruments, specifically the contact mat and force plates, have been used to measure the squat and counter movement jumps (CMJ) [114, 118]. Experts also use common field tests, which do not require expensive equipment, and include the Sargent vertical jump test [119] and the standing long jump [120]. The CMJ involves the participant starting the test in an upright position with his/her hands on the hips and a straight trunk, and then squatting down to a 90-° leg bend, and then vertically jumping [121]. The squat jump involves the participant squatting down to a 90° bend in the knee with hands on the hips and a straight trunk. Once the participant is at a 90° angle, the instrument is reset and the participant vertically jumps [90]. The Sargent jump test compares the maximum standing vertical reach with the maximum jumping vertical reach [119]. The standing long jump involves the participant performing a two-footed horizontal jump, and using swinging arms and bending the knees to produce momentum. Performance is measured as the distance from the starting position to the back of the heels at landing as long as the participant has not fallen or stepped backwards [122].

Studies have reported that all of these tests are valid and reliable measures of explosive power, but the counter movement and squat jumps are the most valid and reliable when measured using a contact mat [90].

However, these tests do not consistently assess the same measures of power. The standing long jump measures power that moves in a horizontal motion, while the other tests measure power in a vertical motion. To best understand changes in explosive power, it is important to measure both vertical and horizontal motions. Although explosive power is important in athletic settings, it is not normally assessed in occupational settings. However, there may be more testing in occupational settings as there is more research conducted to assess explosive power through jump tests in military settings. A study on women in the military found that a loaded squat jump was related to load carrying tasks, which military personnel may be required to perform [123]. This research shows that assessing power through jumps is related to occupational tasks and may justify their utility in assessing the capabilities of military personnel. Research that sought to establish normative data for military personnel in the U.S. used a single leg vertical jump, a 6-m timed hop and a triple hop and found that males performed better in power than females and those who were under 30 years old performed better on power than those over 30 years old [124]. A vertical jump test was also employed in a study on the Australian army special forces for developing the minimum standards for a pre-selection physical capability assessment [125]. A review of the methods that could replace or supplement the current test among U.S. Navy personnel found that the standing long jump was highly correlated and could be used as part of the navy’s physical readiness test [126]. Future research should standardize power testing to allow for comparisons across military populations.

#### Agility

Agility has been defined as a change of direction or velocity through an entire body movement in response to a stimulus [127]. Agility is essential for allowing individuals to change direction quickly without losing balance [128]. Agility has been shown to be affected by several components, including balance, coordination, speed and power [129]. Improved agility leads to benefits, such as increased intramuscular coordination, increased body control during fast movements and a decreased risk of injury or re-injury [130]. Research on possible physical fitness tests for the U.S. Navy found that there is a positive relationship between agility times and jumping ability such that the higher an individual can jump in a single leg jump, the faster their agility times [126]. This indicates that even though not all physical fitness elements are positively related to each other, an increase in performance in one element may automatically lead to an increase in performance in another area. Agility can be very important in occupational settings, specifically in confined spaces, such as on a ship, where changes of direction frequently occur.

Several tests have been used to assess the agility of individuals and teams in athletic and occupational settings. Agility tests can be composed of multiple elements, including acceleration, deceleration and retropulsion, which occur during multidirectional, bidirectional or unidirectional movements [131]. The most commonly used agility tests include a pro agility shuttle [126], *t*-test [132–135], Edgren side-step test [135], zig-zag test [134], the Illinois agility test [134, 135] and the 505 agility test [127, 136–138]. All of these tests are suitable for using in the field because they do not require a lot of space or expensive equipment. However, certain equipment or facilities can make the results of these tests more accurate and reliable. For example, when using time as a measure of performance, timing gates rather than stop-watches could be used to prevent human error or reaction times. In addition, using an indoor non-slip surface rather than an outdoor space could prevent the effect of the weather or floor surface on participant’s performance, and, therefore, enable a better test-retest reliability. Although agility tests are not currently conducted as part of the yearly physical fitness evaluation, studies have assessed agility in military populations. Some military research includes agility under the heading ‘mobility’, which is an essential element of effective movement around different terrains [139, 140]. A study on U.S. Army personnel found that the *t*-test, the side step test and the Illinois agility tests were all valid and reliable measures of agility and, therefore, assess mobility [135]. Additional studies on assessments in the U.S. Army and Navy found that the pro agility test can identify changes in military fitness over time and could be used as part of the physical fitness testing process [126, 140].

#### Muscular strength endurance

Muscular strength endurance has been defined as the ability of the skeletal muscles to perform repeated contractions for an extended period of time. Lower and upper body muscular strength endurance is important because it enables load carrying occupational tasks and carrying casualties during an emergency on-board the naval vessel [2]. There are multiple ways to test muscular endurance and strength, including separate testing for both the upper and lower body. However, when completing a battery of tests, the lower body is often tested with several methods, which include aerobic tests, such as the 2.4 km run [91]. Performing a lower body muscular endurance test may significantly fatigue the lower limbs and impact the remaining testing procedures. In contrast, the upper limbs are used to generate momentum and are not generally taxed unless through specific tests.

Tests for upper body muscular strength endurance include the bench press [141], chest press [141], shoulder press [141], push up [2], pull up [120], biceps curl [142] and grip strength [143]. Although all of these tests have been used in different populations, several, such as the chest, shoulder and bench press tests, require specialized equipment. These tests also use weights, and the subject may need to have several turns before using the correct weight. Additionally, the subject’s body weight is not supported during these tests, thus, they may be biased against those who have a lower body weight and, therefore, lower muscular strength endurance. In contrast, using body weight to test for muscular strength is biased against those who have a high body mass [91, 144], however; the tests may be good indicators of how individuals will perform in the work place where they are constantly required to carry their own body weight.

In military settings, the push test is used to assess upper body muscular endurance [83, 85, 86]. There are several different protocols that can be used during push up tests, including the maximum number of repetitions, push-ups to an audio signal or timed push-ups. The Irish Defence Forces version uses the number of repetitions that can be completed in a set period of time [83]. This test is a valid measure of upper body muscular strength endurance [91].

Pull ups have also been viewed as a valid indicator of upper body strength, however; they target different muscles than push-up tests [145, 146]. When an individual cannot complete chin ups, alternative tests are administered and include the flexed arm hang, in which the participant hangs for a certain number of seconds rather than completing repetitions [147].

The grip strength test uses a dynamometer and is an isometric test of strength that is not indicated as a measure of strength endurance [148]. However, this test can provide valuable insight into upper body strength and measures certain task performance, such as opening a bulk head door, which is necessary on-board navy vessels [2]. Therefore, when tasks, such as opening a bulk head door, are completed on a daily basis, there may be a positive change in grip strength over a prolonged period of time at sea.

The sit up test is also used to measure strength endurance and is part of a battery of tests that highly correlates with casualty carrying, which is one of the occupational tasks that may be conducted in a naval setting [2]. Sit ups are also currently used as part of the fitness test requirements for the navy. They are measured by the number of sit ups that can be completed [83].

Although both sit up and the push-up tests are used in the Australian Defence Forces [149], the U.S. Naval Service [85] and the Irish Defence Forces [83] differ slightly in their protocols. The Australian Defence Forces test for the number of repetitions, while the U.S. Naval Service tests for the number of repetitions in a two-minute period and the Irish Defence Forces test for the number of repetitions in a one-minute period. Additional research is needed to better understand these differences.

#### Speed

Similar to agility, speed is related to mobility, which is part of the required physical capabilities that are needed for a physically fit service member [139]. Although agility has been defined as an individual’s ability to quickly change speed and direction, it does not directly measure an individual’s maximum speed over a certain distance because agility tests require both acceleration and deceleration. Maximal speed is the maximum velocity at which an individual can sprint [150]. Therefore, to measure an individual’s maximal speed, evaluations use a straight-line test that specifies a certain distance that depends on which physical speed capabilities are being tested. Speed testing has been evaluated in studies to ascertain its use as a functional military test. Research on differences in performance between loaded and unloaded sprint times in military personnel used a 30 m sprint to show that when military personnel are carrying more weight, their sprint time significantly increases [151]. More than 50% of this increase occurred during the first five meters, which indicates that a split measurement of speed was recorded [151]. Split times were also used in a study that analyzed the impact of body composition on physical fitness tests in the Croatian Navy [152]. In this study, the amount of time taken to complete five, ten and twenty meter distances showed that a higher body composition had a negative impact on the time taken to complete each distance [152]. A twenty meter sprint speed test was also used in a study on the Brazilian army to assess the effect of strength training on sprint speed test results [153]. There is a lack of research on the most applicable distance for testing Navy personnel’s maximal sprint distance, however; split sprint distance times can provide valuable information about the ability to accelerate. As such, given the short spaces on-board navy vessels, and the short distances that would be completed on-board, the ten and twenty meter distances may be suitable for assessing acceleration time and maximal sprint speed distances. Speed training over a period of eleven weeks can positively affect an individual’s jumping power, jumping height, jumping length, squat strength, sprint speed and agility [137]. Additionally, specific strength training can positively affect sprint test results [153]. This research implies that training physical fitness components can have a positive impact on performance in separate physical components. However, although straight speed training will improve straight speed test results, a combination of speed training may be needed to improve changing direction [154]. In addition to requirements for separate training, separate testing may be important as research has indicated that acceleration, maximum speed and agility are separate elements that are not related to each other and should be tested as separate elements [150].

#### Anaerobic testing

Anaerobic capacity has been defined as the maximal amount of adenosine triphosphate that can be resynthesized via anaerobic metabolism, which includes both lactic and alactic systems, during a specific mode of short-duration maximal exercise [155]. The maximum accumulated oxygen deficit (MAOD) is the most effective way to test anaerobic capacity [156]. MAOD is sensitive to anaerobic training, is highly correlated with high intensity efforts and has been used to validate other methods of anaerobic conditioning. However, calculations require assessments of several sub maximal exercise bouts, one supramaximal exercise bout and a measure of VO_2_ during these bouts. Obtaining these measurements would require a laboratory setting and expensive equipment. Additional field methods have been created to test the anaerobic capabilities of large groups, such as an athletic setting. These tests include the Wingate test [157], the 5-m multiple shuttle run test (5-m MSRT) [158], and the running based anaerobic sprint test (RAST) [159]. Each test has been assessed for reliability and validity across settings. The Wingate test assesses peak anaerobic power and uses a bicycle ergometer [157]. Although several studies have found that the Wingate is a valid and reliable predictor of anaerobic power [160], research also suggests that a familiarization session and a practice session are needed to obtain a valid indicator of anaerobic power [161]. In many field settings, these familiarization sessions are a large disadvantage, as time with participants is often limited. This test may also be unsuitable for testing large groups because specialized equipment is required to complete the test and a recorder for each individual is needed, thus, only one person can be tested at a time. The RAST was developed as a running-based anaerobic sprint test that is similar to the Wingate test in that it measures the maximum power output using weight as one factor, however; this test is more suitable for runners [159]. The RAST requires a track or sports hall that is larger than 65 m with two individuals monitoring the test, which makes it challenging to assess more than one person at a time. Furthermore, although some studies have found that the RAST is a valid method for assessing anaerobic power [162], research also found that it does not predict anaerobic capacity in running when it is compared with results from the MAOD [163].

Another test to evaluate anaerobic capacity is the 5-m MSRT. This test analyzes anaerobic capacity as well as repeat sprint ability and resistance to fatigue [158, 164, 165]. This test efficiently measures the anaerobic energy system and is sensitive enough to detect major changes in fitness over time [164]. There is currently little research on the benefits of including an anaerobic fitness test or which test is the most appropriate to use with military populations.

#### Aerobic testing

The U.S. Department of Defence defines aerobic capacity as the functional capacity of the heart, lungs and blood vessels to deliver oxygen to the working muscles, and it’s use by the muscles to oxidize energy sources [85]. Although researchers have created tests that use a laboratory setting (direct measures of VO_2_max) to examine aerobic capabilities, these tests can be expensive and time consuming to use with large groups in athletic or occupational settings. Several field tests (indirect measures of VO_2_max) are currently used across settings. These field tests include the Cooper 2.4 km run test, which is an adaptation of the original Cooper test [166], MSFT [167] and the Rockport walking test [168], which are currently used in military settings [2, 169, 170]. Additionally, the Harvard and the Chester step tests [171] are both recommended as alternatives to the direct testing of general seafarers [80]. Other field tests include the University of Montreal track test [172] and the Yo-Yo intermittent shuttle run test [173]. Each of these tests should be administered with specific populations in particular environments, thus, it is important to choose the most suitable test.

Several tests have specifically been created for athletic settings. For example, the Yo-Yo intermittent shuttle run test evaluates the physical fitness of soccer players [173]. The benefit of this test is that it assesses an individual’s aerobic and anaerobic ability in one test, and both of these are used during match play in several team sports [174]. However, the disadvantage of testing both aerobic and anaerobic systems during a single test is the inability to differentiate between the two systems. Therefore, to ascertain one’s aerobic ability, it is critical to use a specific aerobic test.

The Rockport walking test involves walking as fast as possible for one mile [168] and meets these criteria as it tests aerobic capabilities. Because it is a walking test, it is only used with individuals who have poor fitness, medical conditions that prevent maximal exertion or who cannot complete a running test on a similar distance [86]. The University of Montreal track test requires the participant to move around a track that is marked at 50 m intervals to the sounds of an audio recording [172]. Studies have shown that this test is valid for assessing both maximal and functional aerobic capacity in moderately trained individuals [175].

As with the University of Montreal track test, the Cooper 2.4 km run test is conducted on a track [2, 166]. Although this test has been used to assess military populations, there is little research on its validity. Most researchers have examined the original version of the test [166], which consists of a 12-min run for distance. The 12-min run has also been used in military settings to assess aerobic conditioning [169]. However, for high test-retest results, it is important that as many variables as possible remain constant. Although this test needs little equipment, it must occur on a track or in a sufficiently large marked area, which often means that the test must be performed outside. Outdoor conditions may affect the results due to weather conditions on any given day. For example, if an individual performs the test on day one in the rain with strong winds and completes it in 25 min and then performs the test a year later when it is sunny with very little wind and completes it in 15 min, it is not clear whether the change is due to gains in physical fitness or the change in climate.

The MSFT, the Harvard step and the Chester step test can all be completed in a sports hall, thus, they are not affected by climate conditions. The Chester step test was developed to predict maximal aerobic power (VO_2_ max) through sub maximal testing in fire brigades and has been used in other occupational settings, including ambulance services and airport firefighters [171]. Although the Chester step test is valid in test-retest ability, it is not clear whether it can predict VO_2_ max [176].

The MSFT has become widely used as a field test in both athletic and occupational settings [177–179]. The test assesses VO_2_ max, which is calculated using the level achieved during the test [167] that is cross referenced with a table of oxygen uptake values [180]. Research originally indicated that this test was a valid indicator of VO_2_ max in active men and women [181, 182]. Additional research found that although the test has a strong test-retest reliability, it tends to underestimate VO_2_ max when compared to a direct measure [183], with improved accuracy depending on the prediction model [184, 185]. Similar to the Chester and the Harvard step tests, this test accounts for a person’s ability to carry their own body weight, however; the MSFT can be influenced by running economy and mechanical efficiency [186], which may be crucial to moving around a ship.

A study on the royal British Navy recommended that all personnel going to sea attain a VO_2_ max of 41/min/kg, which could be assessed with the 20 m shuttle run or 2.4 km run tests [2]. The 2.4 km run relates to level ten and one shuttle for males and level seven and six shuttles for females [187]. However, a study that compared the Cooper 12-min run with the multistage shuttle run test found that the Cooper 12-min run test underestimated VO_2_ max at lower VO_2_ max values and overestimated VO_2_ max at higher values. These over and underestimations were not apparent in the multistage shuttle run test [188]. Although caution should be used when attempting to predict VO_2_ max from field test data, the multistage shuttle run test may to be more useful than the 12 min run because there is a consistent mean bias across fitness levels [188]. As a result, when laboratory testing is not feasible, the MSFT is the most suitable and reliable test for measuring aerobic capabilities, specifically in military settings where it is an appropriate testing method [2].

### Monitoring physical activity

Over the last several years, many studies have examined physical activity monitoring among specific populations. Physical activity has been defined as energy expenditure that results from any bodily movement that is produced by the skeletal muscle [189]. In athletic populations, monitoring ascertains the demands that are placed on the body in specific disciplines and can enable tailored physical, nutritional, injury prevention and rehabilitation programs. It can also be used in general populations to assess health behaviors and their association to current health status, which provides justification for an intervention [190]. This type of monitoring can also be applied to occupational settings and provide valuable information. Physical activity can be measured using both indirect and direct measures. Indirect measures involve self-reported information, such as questionnaires, diaries or logs. This form of data collection can effectively be used in large groups as it is inexpensive and easy for the participant to complete [191]. However, self-report methods may also underestimate or overestimate levels of inactivity, actual physical activity and energy expenditure [190], which questions their reliability and validity [192]. Direct methods can increase accuracy or validate indirect measures. Direct methods include time motion analysis, heart rate monitoring, global positioning satellite systems (GPS), pedometers, motion sensors and accelerometers to monitor individual’s activity levels and physical responses [193, 194]. Heart rate monitors have been used in various settings to monitor the physical demands that are experienced by individuals during different forms of physical activity [195]. Heart rate monitors are useful because heart rate linearly increases with oxygen consumption during moderate to strenuous activity [196] and can evaluate VO_2_ max [197]. Heart rate monitors have been combined with other equipment, such as GPS, to provide more insight into the physical demands and energy expenditures that are experienced at different levels of physical activity [197]. However, heart rate monitors also have limitations for monitoring physical activity. Heart rate can be affected by many factors including stress levels, illness, emotions, temperature and caffeine intake [198]. Therefore, it can be difficult to establish why an individual’s heart rate increases during periods in which the level of physical activity does not change or, when the level of activity is low, the activity or additional stimuli may be affecting the heart rate [199]. Heart rate is usually determined with a heart rate monitor that is held around the chest with a strap. This position can be both uncomfortable and constricting for the wearer, especially when it is worn for prolonged periods of time. Alternatively, when the strap is too loose, heart rate may not be properly detected and there may be errors in the obtained results [199]. Time motion analysis involves video recording an individual or a game that is then analyzed to assess movement patterns and categorized into movements such as walking, running, sprinting etc. [200]. Although this data is useful in athletic settings, it is challenging to collect in occupational settings when the individual moves in and out of different locations. It may also be harder to get permission to use a video in an occupational setting, due to privacy and confidentiality concerns, especially on-board a navy vessel. GPS usually uses a network of 24 satellites that orbit the earth to track the position of a receiver, such as a watch [201]. This type of monitoring is very valuable for individual athletes or team sports as several players can be tracked at once [202]. However, GPS monitoring can only be used on a stationary surface as the satellites cannot differentiate between the movements of the individual and the movements of a form of transport. Therefore, alternative methods need to be used to analyses individuals in occupational settings, such as on-board ships that may be moving.

Pedometers, motion sensors and accelerometers can all be used on-board ships as they do not depend on satellites to monitor movements. Pedometers are used to measure ambulatory physical activity. They use technology, such as spring levered and piezoelectric sensors, to determine how many steps have been performed by the individual [203]. The limitation of these pieces of equipment are that they only measure steps during walking or running and cannot to gauge distance [204]. Pedometers have the lowest level of accuracy when measuring steps at a speed of 2.0mph [203, 205, 206]. In contrast, accelerometers measure movement intensity [207] and can be used during all types of physical activities and inactivity, from sleeping to running. An accelerometer is an electromechanical device that detects and records motion on a single or multiple planes. There are two different types of accelerometers; uniaxial and triaxial. Uniaxial accelerometers record and store acceleration during a specific period of time on the vertical plane allowing for measures of vertical displacement. Triaxial accelerometers record and store data that are measured from acceleration on three different planes. As a result, triaxial accelerometers are more reliable and valid than uniaxial accelerometers, heart rate monitors and hip pedometers for assessing energy expenditure in unregulated [208] and regulated [209] play in children. Accelerometers can also be used to assess energy expenditures during walking and sedentary activities [210]. However, accelerometers have been found to underestimate energy expenditures during static exercise, which may not be a major limitation in free living conditions [211]. It is not clear whether these monitoring devices would be affected by a ship’s normal movement.

## Psychological impact

An individual’s mental health can have a significant impact on overall wellbeing. Mental health problems can affect energy levels, concentration levels, as well as motivation and judgement, which are required for successful performance in military occupations [212]. Most research that investigates psychological wellbeing among military populations focuses on post-traumatic stress or the consequences of deployment into combat situations [213–219]. Although it is a common perception that deployment into combat has a negative effect on mental health, a study on U.K. military personnel who were deployed to the Iraq war found that normal U.K. military personnel did not experience adverse mental health issues [213]. In this study, only reservists who were deployed experienced adverse mental health affects [213]. These findings are consistent with a follow-up study with a similar population that found low levels of possible post-traumatic stress disorder [215]. In contrast, several studies that were conducted with the U.S. military found that those returning from deployment to the Iraq and Afghanistan wars had an increased prevalence of mental health disorders and specifically post-traumatic stress disorder (PTSD) [220–226]. The contrast in these studies indicates that U.K. military personnel are differentially affected by deployment to war than U.S. Military personnel. Research has also shown that the occurrence of common mental health disorders is similar to or higher in the U.K. military than the general population and are not affected by military exposures [227]. This difference may be due to several factors, including shorter operational tour lengths, differences in access to long term healthcare, variations in combat exposure and demographic differences [227]. Research has also suggested that the frequency of mental health problems increases with multiple deployments into war zones [228]. Thus, a higher number of military personnel with multiple deployments in the U.S. than in the U.K. may contribute to higher levels of PTSD. However, Hunt et al. [227] argues that the in the 2003 Iraq war, two-thirds of U.K. military personnel had previous deployment experiences as compared to only 10% of U.S. military personnel, therefore, the U.K. troops were more experienced with combat stressors and could cope better as a result.

Although Irish Naval Service personnel may experience some combat situations over the course of their career, due to a lack of exposure to war conditions, they are unlikely to experience the same post-traumatic stress and psychological issues as other combat focused navy cohorts. However, research has indicated that naval service personnel suffer from similar stressors as general seafarers [229] and that the prevalence of mental health disorders have been associated with adverse working conditions [230, 231]. Seafaring occupations can extensively vary from general land based occupations in the psychological, psychosocial and physical stressors that are experienced at sea [37, 232]. Studies have indicated that working on-board merchant ships can be one of the most physically and mentally demanding occupations, and lead to a potential for severe psychological distress [233–235]. Research on the Brazilian army linked job stress to higher levels of occupational activity and lower levels of physical leisure time activity [63]. Stress has been defined as the response to a situation or event [236]. It involves a situation that the individual perceives as important for his/her well-being and where the demands are greater than the coping resources [236]. Research on seafarers found that the risk for stress is increased during journeys that have a long duration and during the night [237]. A study on German seafarers found that of twenty-three stressors rated by seafarers; time away from family, time pressure/hectic actives, long working hours per day, heat in work places and insufficient qualifications of subordinate crew members caused the most stress [65]. Although some of these stressors may be unavoidable, such as heat in the work place, other stressors could be avoided. However, stressors, such as long periods of time away from family and friends, appear to be a growing problem [238]. This social isolation may have become a greater problem due to a decrease in crew sizes and the amount of time spent in port, which resulted from faster turnaround times [239]. These issues appear to be easily resolved, however; this would require financial investments that navy or seafaring organizations may not be willing or able to spend.

Stressors, such as fatigue, may have some physical warning signs, however; many other stressors may have a large mental impact on the individual without obvious warning signs. Research on the general seafaring population found that over 5.9% of deaths at sea resulted from suicide [240]. This research indicates that seafarers who experience mental health issues may not be willing to seek help, which could result in fatal consequences. A study on PTSD in the U.K. found that a significant proportion of individuals who develop PTSD are not willing to seek help and only search for help when persuaded by others to do so, which may increase the prevalence of delayed onset PTSD [241]. Research has indicated that there is a reluctance to obtain professional help for a mental health problem in both military and seafaring populations [229]. This reluctance may be because seeking help may lead to a loss of medical certification, which would result in a loss of work [229]. This may also be due to the stigma that is associated with mental health problems [229], which was shown in U.K. ex-service personnel, who named embarrassment as a barrier to seeking help [242]. Research on U.S. soldiers and marines found that individuals did not seek help due to a fear of being perceived as weak or being treated differently by unit leaders [243]. This fear was also found in the U.K., Canadian, Australian and New Zealand Defence Forces [244]. Research on the Brazilian army found that those who suffer from psychological distress also have a high prevalence of leisure time physical inactivity [63]. This type of inactivity may lead to several physical medical problems, such as obesity, which can, in turn, affect individual’s occupational ability.

### Psychological measurements

In the Irish Naval Service, each individual is asked to fill out a Defence Forces Medical Questionnaire prior to their annual medical assessment. As part of this questionnaire, the individual responds to five questions that refer to mental health. The five questions include topics such as depression, suicide, trouble sleeping, loss of interest in hobbies and irritability. If these questions are satisfactorily answered, then no further action is needed. However, if the medical practitioner is alerted to an issue as a result of the questionnaire or the subsequent examination, then the individual may be referred to a defense forces psychologist. Some studies believe that a full evaluation of mental readiness should be performed with each individual prior to deployment to prevent those at risk of harming themselves and others from going to sea [229]. It may also be of benefit to use these screening methods before and after basic training. This screening could allow for the early detection of mental problems and prevent those who are unsuitable from joining the navy. To provide this type of screening to large groups of individuals in a short time period, additional psychological screening tools may be required. There are several methods that researchers have used to measure psychological wellbeing in occupational settings or among a particular population. A significant amount of these methods require that the individual completes a questionnaire. In relation to military and seafaring populations, questionnaires include: the Primary care evaluation of mental disorders patient health questionnaire (PHQ) [214, 245, 246], the Kessler psychological distress scale [247, 248], the post-traumatic stress disorder checklist-civilian version (PLC-C) [213, 248–250] and the General Health Questionnaire (GHQ-12) [213, 229, 251, 252].

Due to the lack of exposure to combat and the suspected low levels of PTSD in many naval populations, the PLC-C may not provide insight into the mental health of naval service personnel. Instead, a more general mental health questionnaire may be required. The Kessler psychological distress scale was designed to differentiate between cases of serious mental illness and non-cases and consists of either a ten question or six question scale [247]. Although this scale is a useful tool, it may not provide an indication of general mental health but may reveal whether an individual is suffering from a serious mental condition [247]. The general health questionnaire was created in 1978 to assess mental wellbeing [253]. It can be used to detect mental problems, such as somatic symptoms, social withdrawal, anxiety and depression.

The GHQ originally had sixty items, however; due to time constraints thirty, twenty-eight and twelve item scales have also been created [254]. Research found that the GHQ-12, which is the twelve item version, is just as reliable and valid as the longer version and takes less time to complete [255]. This questionnaire has been used in several military [213, 248, 252] and in particular naval service populations [229, 256], which indicates its value for providing knowledge about these populations.

Although stressors may differ according to the situation, both combat and non-combat navy cohorts regularly need to cope with varying stressors on-board [229]. As such, research is needed to ascertain how navy personnel cope during stressful situations. To measure this coping ability, it is important to use questionnaires, such as the coping inventory for stressful situations (CISS) [257, 258]. This questionnaire is a self-reported questionnaire that was designed to assess three dimensions of coping strategies that are used during stressful conditions [257, 259]. It consists of 48 questions that assess avoidance (which is further reduced to distraction and social diversion), emotion and task orientated coping styles [257]. Each question is answered on a scale that ranges from one to five, with one referring to them never doing this in a stressful situation and five referring to them normally doing this in a stressful situation [257]. This questionnaire excellent internal consistency and good to adequate test-retest reliability [257].

Research has shown that the ability to cope with stress is related to an individual’s personality, and because personality is hard to change, coping mechanisms are consequentially difficult to change [260]. Establishing a relation between the ability to cope with stress and personality traits in naval service cohorts would allow organizations to choose individuals who are well suited for the most stressful occupations on-board a vessel. Although there are several personality questionnaires, the most commonly used is the big five personality test. The big five personality test was originally designed to assess an individual’s personality traits [261]. Researchers have developed several versions of the big five personality test [262–265]. One of the most commonly used versions with military and seafaring samples is the NEO five factor inventory (NEO-FFI), which asks the individual to respond to one hundred and twenty statements [266–269]. The NEO-FFI measures an individual’s personality traits in relation to the following elements: conscientiousness, extraversion, openness to experience, neuroticism and agreeableness [267]. Participants rate sixty statements on a five-item scale from strongly disagree to strongly agree. This test has acceptable levels of reliability and validity in military populations [270, 271].

## Fatigue

Fatigue has been defined as ’a reduction in physical and or mental capability as the result of physical, mental, or emotional exertion which may impair nearly all physical abilities including: strength, speed, reaction time, coordination, decision making, or balance” [272–274]. Fatigue has been shown to increase anxiety, decrease vigilance and negatively affect work capability and efficiency [275, 276]. Fatigue results from a lack of adequate sleep. Research has found that the increase in lack of sleep leads to an increase in micro sleeps, which are involuntary sleep periods that last between half a second and ten seconds [275]. Research has shown that fatigue can have a serious impact on individuals across environments including in occupations such as mining [277].

Fatigue has also been identified as a serious occupational risk on-board ships [272, 278] and is a major factor in accidents on-board ships [279–281]. However, unlike in other occupations, it can be much harder for seafarers to escape from or change their environment. There are many different factors that contribute to seafarer’s experience of fatigue. Research has identified that fewer personnel and longer working hours are the main factors that lead to crew fatigue [65]. Studies have indicated that fewer personnel resulted from the automation of various tasks, budget constraints and the improper scheduling of work/rest hours [282]. The Maritime Labour Convention (2006) states that all ships must have an adequate number of employees on-board to ensure that the ship is run efficiently and safely [283]. However, this convention has not prevented a continued decrease in the number of crew members [37]. In the U.S. Navy, the navy standard workweek model (NSWW) outlines the number of hours a sailor will work/rest on-board ships. However, a postgraduate study that used self-report data from sailors found that 61% of participants exceeded their 81 h of allowed working time [284]. On average, working hours were exceeded by over 20 h per week, which also indicates that participants got nearly nine less hours sleep per week than is recommended in the NSWW [284]. Similarly, two other post graduate studies on different vessels in the U.S. Navy found that participants worked more hours per week on average than allowed by the NSWW and that they suffered from inadequate sleep [285, 286]. Research on the Australian Navy also found that fatigue was an issue and that 44% of participants reported working over 80 h per week, while 62% reported not getting enough sleep [287].

Although sleep requirements and habits may vary according to the individual, all people require periods of unbroken sleep [37]. Research has shown that adults require approximately eight hours sleep to offset sleep debts and achieve optimal performance [288]. The environment in which a seafarer works and lives can be much different than those that land based workers experience. While trying to sleep, the seafarer must address factors, such as the noise and vibration of the ship, and adverse weather conditions that can cause irregular ship movements [37]. A study on the royal Norwegian Navy found that noise levels on-board vessels exceeded the recommended amount, which could lead to adverse health effects [289]. Research has also found that sleep disruption that results from noise can vary according to the individual’s age and the location of their sleeping quarters [65]. Younger individuals tend to be more prone to sleep complaints because they are more sensitive to noise [65]. This research indicates that younger individuals should be located in quieter sleeping areas on-board vessels to ensure proper sleep and aid in fatigue prevention. Research indicates that working shifts can lead to a lack of sleep and result in a sleeping disorder that is known as shift work sleep disorder (SWSD) [290].

Evaluations of sleep duration and sleep quality at sea found that, on average, watch keepers had a lower sleep duration than those who worked day shifts [291, 292]. Additionally, research on to merchant marine personnel found that those on watch keeper shifts had critical fatigue levels as often as 24% of the time [293]. Research that examined traditional shift workers found that the timing of the shift can influence the amount of sleep [294]. This finding has also been shown at sea, where those on the watch keeping shift from 04.00–08.00 get less sleep than those on alternate watch keeping shifts [293]. Individuals on this shift obtain less than four hours of sleep per 24 h period approximately 22% of the time [293]. The negative effect of shift work could result from disturbances to circadian rhythms [295], which are the manner in which the body operates with attentional peaks and troughs at various stages throughout a 24 h period [233]. Along with the type of shift worked, the on-board occupation can have an impact on sleep quality. On merchant vessels, third officers reported the lowest duration and quality of sleep [292]. A report on Australian seafarers found that pilots and engineers reported a higher incidence of poor sleep quality than the deck crew and master/mates [296].

A study on the royal fleet auxiliary used the need for recovery scale and found that individual’s frustration on-board the ship was related to work related fatigue, and that this fatigue accumulated over time when continuously exposed to work demands on-board [297]. Research has also indicated that a lack of sleep can lead to increased food intake and weight gain, which can result in obesity [298, 299]. Reducing frustration levels and weight gain may lead to benefits, such as personnel retention and increased job satisfaction. This may be achieved by simply ensuring that each individual on-board the ship receives at least eight hours sleep on most nights, or that individuals should work shifts that suit them [233].

### Measurements of sleep

Both a lack of sleep and sleep disorders have become widely researched because of their serious consequences on the human body [290, 300–302]. Over 80 different sleep disorders have been identified by the International Classification of Sleeping disorders [303]. A polysomnogram (PSG) is currently the gold standard for sleep monitoring. This usually involves monitoring the individual’s sleep patterns with the electroencephalogram, electrooculogram, electromyogram, electrocardiogram, air flow, thoracic and abdominal movements, and oximetry during an overnight period [290]. However, a laboratory setting is required to perform a PSG in addition to specialized equipment and a sleep specialist, all of which are expensive and may be in short supply [290]. Therefore, to monitor sleep in a specific environment there is a need for alternative methods. The most common form of data collection in relation to the sleep habits of military personnel are questionnaires. Questionnaires include the Pittsburgh sleep quality index [304], sleep disorder scale [305], Epworth sleepiness scale [306], the Bergen insomnia scale [307], the Stanford sleepiness scale [308] and other self-report methods [309]. As already discussed, a lack of sleep can cause many issues, especially on-board navy vessels. For example, disturbances of sleep among navy personnel in the Singapore Navy, which were monitored using the Stanford sleepiness scale, had a negative effect on perceptive abilities, cognitive abilities and mood [308].

The predominant use of questionnaires in military populations suggests that the results are measuring a perception of lack of sleep. However, sleep is subjective, and while six hours’ sleep might suffice for some individuals, others may need eight or nine hours sleep to feel rested. Although questionnaires can indicate how an individual is feeling based on their sleep patterns, they may not provide an actual indication of how many hours of sleep were obtained. To assess actual hours of sleep, researchers have used physical measures to monitor sleep patterns, such as cardiovascular, respiration, audio, actigraphy, body position and temperature [290]. These measurements have been combined in several ways to increase the accuracy of the results. For example, multi-sensory devices combine an accelerometer, skin temperature, galvanic skin response and heat flux from the body. This form of measurement is a reliable method for analyzing sleep in individuals with obstructive sleep apnea [310]. Sleep monitoring of seafarers through using accelerometers has become more common over the last several years [311, 312] and may enable comparisons of sleep patterns across different populations.

## Nutrition

As previously discussed, obesity is an increasing and costly problem for those working in occupations at sea as well as for the military. This issue occurs due to several factors that include a sedentary lifestyle and the nutrition that is available on-board ships. The 2006 Maritime Labour Convention outlines the requirements for living conditions and the provision of food on-board ships. It states that its purpose is, “To ensure that seafarers have access to good quality food and drinking water provided under regulated hygienic conditions. 1. Each Member shall ensure that ships that its flag carry on-board and serve food and drinking water of appropriate quality, nutritional value and quantity that adequately covers the requirements of the ship and takes into account the differing cultural and religious backgrounds. 2. Seafarers on-board a ship shall be provided with food free of charge during the period of engagement. 3. Seafarers employed as ship’s cooks with responsibility for food preparation must be trained and qualified for their position on-board ship” [313]. Although this convention aims to ensure that sailors receive adequate nutrition during service, it also gives sailors less control over their nutrition on-board when compared to on land. Research indicates that seafarers believe that food on-board is very important and can impact both wellbeing and job satisfaction [314]. Similarly, a study on U.S. Military personnel found that diet had more of an impact on being overweight than a lack of exercise [11]. Additionally, only 13% of military personnel consumed three or more servings of fruit and only 14% consumed three or more servings of vegetables per day [11]. These figures were less than the rates of daily consumption for civilians [11]. On-board a ship, differences in consumption could be caused by the lack of available foods that have nutritional content, and could lead to malnutrition. In the U.S. military, the symptoms of eating disorders are common and could result from the need to be at a certain weight in the military or because those who are more at risk for eating disorders chose to join the military [315].

Most of the responsibility for the food on-board seafaring vessels lies with the on-board chefs. A study on Danish seafarers found that one of the food preparation challenges on-board ships is that many of the cooks have very little training and had limited cooking abilities [316]. The same study also found that a lack of storage and equipment as well as a low frequency of supply options were challenges to on-board food preparation [316]. In addition to these challenges, seafarers who work shifts may eat at irregular meal times [6], which can disrupt regular eating habits. Food is required as a source of fuel for the body, however; the over or under consumption of food can lead to issues, such as obesity, malnourishment and anorexia. Malnutrition can lead to depression in immune function [317], reduced physical performance [318] and prolonged recovery from injury and illness [319]. Therefore, it is important that individuals consume an adequate amount of food to ensure nourishment without consuming too much that it results in obesity. Controlling consumption levels is necessary in confined occupational spaces, such as on-board ships, where energy expenditures may significantly differ from those on land due to a lack of time or facilities for physical activity. However, to gain an understanding of the impact that nutrition has on individuals on-board a ship, it is important to understand the energy that is consumed by individuals compared to the energy that is expended. The standard unit of energy in the metric system of measurement (SI) is the joule. Under European law, it is required that all food labels display energy in kilojoules (kJ), however; the most commonly known measurement of energy is the kilocalorie (kcal) [320]. The Calorie is defined as the amount of heat required to increase the temperature of 1 kg of water by one degree [320]. The Calorie is used on food labels as an indicator of the potential energy in foods and the chemical energy that is stored in human tissues that can be removed by work [320]. Older research indicated that seafarers in the merchant fleet had an average daily energy consumption of 3,000-3,500 kcal [321]. This figure could vary according to weather conditions or the level of physical work conducted [322]. More recent research found that hot weather conditions did not affect energy expenditures, however; energy expenditures increased during cold weather conditions [323]. In contrast to the previous research on seafarers [321], a study on royal marines who were deployed to Iraq for 6 months found that the estimated daily energy expenditure was higher at approximately 3,625 kcal per day and that the estimated daily energy intake was 2,531 kcal [324]. Research that investigated the US army during training exercises found that individuals had an energy expenditure of approximately 4,207 kcal per day [53]. This figure exceeds the figure of 3250 kcal that is outlined as the military dietary reference for energy intake [53]. In both of these studies, participants had deficits of energy during the testing period. This deficit can lead to a decrease in mean body mass or a reduced ability to affectively perform occupational tasks [324].

An overview of the research on seafarers found that the food patterns of seafarers has not been comprehensively studied and that more research is needed in areas such as energy consumption [6], which is also true for navy populations. When issues such as obesity or the prevalence of eating disorder symptoms can be identified as a result of this research, then it would be essential to implement intervention or prevention programs [325, 326].

### Measurements of energy expenditure and consumption

Calculating both energy expenditure and energy consumption can be very valuable to researchers and individuals. When the calculation of energy expenditure is feasible, the individual can consume an adequate amount, which can prevent obesity and other factors from becoming an issue. Researchers have used several methods to measure individual’s energy expenditure. These methods include direct or indirect calorimetry [327] and doubly labelled water [328]. Direct or indirect calorimetry involves calculating the metabolic cost from oxygen consumption. To use this method, the participant must be attached to equipment that can collect expelled air, such as a respirometer [323]. Although this method is reliable, it is difficult to test large groups of people and not feasible to test energy expenditure in an occupational setting, such as on-board a navy vessel due to the need for specialized equipment.

The doubly labelled water method involves the complete or partial replacement of both hydrogen and oxygen in water with other elements, such as deuterium or oxygen-18. After a period of fasting, the participant ingests the doubly labelled water and samples of urine, blood or saliva are periodically collected to measure the metabolic rate [323]. Although this method has been validated in certain military populations [53, 324], it may not be appropriate for predicting energy expenditure on-board a military ship where access to and the collection of urine, blood or saliva may be quite difficult.

Direct or indirect calorimetry and doubly labelled water are the gold standards for measuring total energy expenditure but are expensive and need specialized equipment and facilities. As a result, researchers examined alternative methods for assessing energy expenditure, such as the intake-balance method [329], the factorial method [330], pedometers, accelerometers and multi-sensory activity and lifestyle monitors. The intake-balance method uses the estimated food intake and changes in body composition to estimate energy expenditure [323]. To obtain accurate results, a long evaluation period is required and the results rely on accurately estimating energy intake [329]. The factorial method involves the participant recording the duration and the type of all physical activities. Then, the total energy expenditure is calculated using published literature that shows the energy expenditure for a specific recorded activity or one that is similar to the activity [323]. However, this method can be inaccurate as it relies on research that greatly varies [323]. The participant must also record all performed activities, which may not be feasible in an occupational setting [323]. Furthermore, in specific occupational settings where no research has investigated energy expenditure, it may be challenging to accurately compare daily occupational activities to research that already exists.

Alternatives to these methods include pedometers and accelerometers, however; more recent technology has integrated pedometers or accelerometers into multi-sensory devices. Devices that combine physiological and mechanical methods in a single device can increase the accuracy of estimating the energy costs of physical activity [331]. A study on adults in free-living conditions found that such devices have the potential to accurately assess daily energy expenditures compared to the doubly labelled water method [332]. Similarly, additional research has found that devices can accurately estimate daily energy expenditures during rest and low to moderate intensity physical activity in adults [333–335]. However, research has shown that during high intensity exercise, an underestimated energy expenditure can occur with these devices [333]. In an occupational setting, this underestimation may not be a problem unless the individual engages in high intensity leisure time activities that could lead to an underestimation of the total daily energy expenditure [333].

By using food labels and weighing equipment, it would be possible to calculate the amount of energy consumed from each bite of food that is eaten. This method can be very onerous, and on-board ships where individuals are not involved in the food preparation process, it is not feasible. An alternative method is food frequency, which assesses food consumption patterns over a long period of time [334]. However, this method does not evaluate factors such as the quality of the food that is consumed or different meal patterns [334]. In military populations, researchers have estimated energy intake with food diaries [324, 335]. A food diary involves the participant recording the food and drink that was consumed over a specific period of time. Once the food diary has been completed, it is entered into a nutritional analysis program that evaluates the energy intake for particular meals or periods of time. Although food diaries can useful for measuring energy intake, their accuracy may be affected by the participant’s recording ability [336].

## Substance abuse

As discussed in the previous section, both seafarers and military personnel suffer from mental health issues that can result from their occupational conditions. These mental health problems, as well as other occupational factors, such as deployment to combat zones [337], may be related to the prevalence of substance abuse in these populations. The most common types of substance misuse are tobacco and alcohol misuse. Research in both the U.K. and the U.S. found a positive relationship between alcohol misuse and combat deployment [227, 338]. This relationship also increased over time and was particularly high in those who thought they might have been killed or had experienced hostility during combat deployment [337]. Similarly, a three and a half year follow-up study on individuals in the Sri Lankan Navy who had served in combat areas found that after three and a half years post combat, levels of hazardous drinking increased amongst regular forces [339].

Although levels of alcohol consumption are high in several military populations, these high rates have been related to deployment in combat zones. However, studies that investigate general military populations in the U.K. [340] and the U.S. [11], and not just combat deployed populations, also found high levels of alcohol consumption. A U.S. Military survey found that alcohol consumption levels are higher compared to the general population and that this increase could, in part, be attributed to the high percentage (47%) of males aged eighteen to twenty five in the population [11]. However, the levels of excessive alcohol use, which are defined as consuming five or more standard drinks per typical drinking session, at least once a week, have remained relatively constant between 1980 and 2008 [341, 342]. This research may indicate that the issue of excessive alcohol consumption has existed for many years but may not be increasing.

Research in the U.S. Navy found that individuals also drink alcohol because they expect that it will alleviate stressors [343]. Because seafaring and military occupations are physically and mentally taxing, this stress could contribute to excessive alcohol consumption in these populations. In contrast to the high levels found in the U.K. and U.S., research on the Australian Armed Forces found that the prevalence for alcohol abuse was lower for the armed forces compared to the general population [344]. Additional research on the Australian Defence Forces found that alcohol disorders were the most prevalent in the navy [248], which indicates that this population may be at a higher risk. However, there is a distinction between alcohol disorders and alcohol consumption. Those who drink to excess may not necessarily be diagnosed with an alcohol disorder. Excessive alcohol consumption is common in military personnel [345], with binge drinking rates around 43% in U.S. Military personnel [245, 246, 346]. Therefore, although the levels of alcohol abuse may be low in the Australian Defence Forces, rates of alcohol consumption or binge drinking may be high. Without further research and a clear distinction between excessive consumption and an alcohol use disorder, it is difficult to understand the severity of alcohol misuse.

Research has shown that smoking can have a detrimental effect on an individual’s health. Not only has smoking been linked to various forms of cancer and other medical conditions [347], but it also affects an individual’s fitness and productivity levels [348]. In addition, smokers have reduced mental capacities, fitness for duty [349] and readiness and are more inclined to have substance abuse and legal issues [350, 351]. As with alcohol, the rates of smoking are high among military populations [352–356]. In contrast, U.K. soldiers had the lowest prevalence of smoking, with 31.3% of the population being current smokers [353]. Research on the U.S. Armed Forces found that the level of current smokers was higher for younger service men than their civilian counterparts and similar for older service men compared to their civilian counter parts [357]. A study on the Kingdom of Saudi Arabia military found that naval service personnel had the highest levels of smoking of all of the military sectors [358].

Research has examined several reasons for high levels of smoking among military populations. One reason is the effect of deployment in combat zones [359]. Research on the Sri Lankan Navy found that although the prevalence of PTSD was reduced three and a half years after combat, there were increases in rates of smoking among both regular and special forces [339]. This study could indicate that although PTSD symptoms could be reduced after this period of time, other underlying mental issues may remain and individuals may cope with these issues by using alcohol and tobacco. In the U.S. Military, smoking is estimated to be high because it is a part of the military culture that may support and even encourage military members to smoke [360]. Similarly, in the Nigerian military, peers influence whether individuals smoke [361]. Smoking has also been found to be used in military settings as a means of coping with anxiety and a lack of sleep [361]. Because the fatigue that is caused by a lack of sleep is an issue in navy populations [287], addressing sleep deprivation may have a possible influence on rates of tobacco consumption.

Although both alcohol and smoking can be detrimental to an individual’s health they may cause even more health issues when they are consumed together. A study on the Sri Lankan Navy found that current smoking was strongly related to current alcohol use [359]. In the U.S. Military, during the six-week period of basic training, the use of any tobacco or alcohol is forbidden [362]. After this period of time, the number of smokers was reduced by 23.7% [362], which may indicate that bans may be a good method for encouraging smoking cessation [363] and may affect the levels of alcohol consumption. For alcohol consumption in the U.S. Military, policy directives aimed to reduce substance abuse have been implemented since 1972 [364]. However, despite these directives, rates of alcohol consumption and abuse have remained steady, which indicates that the policies have been ineffective [10]. Because there is no research on the Irish Naval Service for levels of alcohol consumption or tobacco use, it is impossible to compare this population with other military cohorts. More research is needed to establish the current levels of both tobacco and alcohol use or abuse as well as their association with each other before implementing cessation encouragement methods.

## Future directions

Although research has been conducted in relation to the various aspects of life within the naval service, most of this research tends to be either retrospective or only targets a small research area. In order to allow organizations to proactively deal with issues that may be faced by individuals within the naval service, future research should look at every aspect of lifestyle and the environment experienced by individuals throughout their career. This should start with how individuals are recruited and trained both physically and psychologically to deal with life in the naval service. Research could help to ascertain if certain individuals, physical fitness levels or psychological traits are more suited to specific occupations within the naval service. Physical fitness testing for occupational readiness is currently targeted at specific physical fitness components, namely strength and aerobic conditioning; however, there is a lack of research to support the occupational relevance of these tests. Research should analyze other methods of assessing physical fitness and ascertain if occupationally relevant tests can be created. Similarly, body composition analysis methods should be tailored towards the naval service and away from the use of generic forms of analysis such as the BMI which may not be suitable for us with a naval service population.

**Fig. 1:**
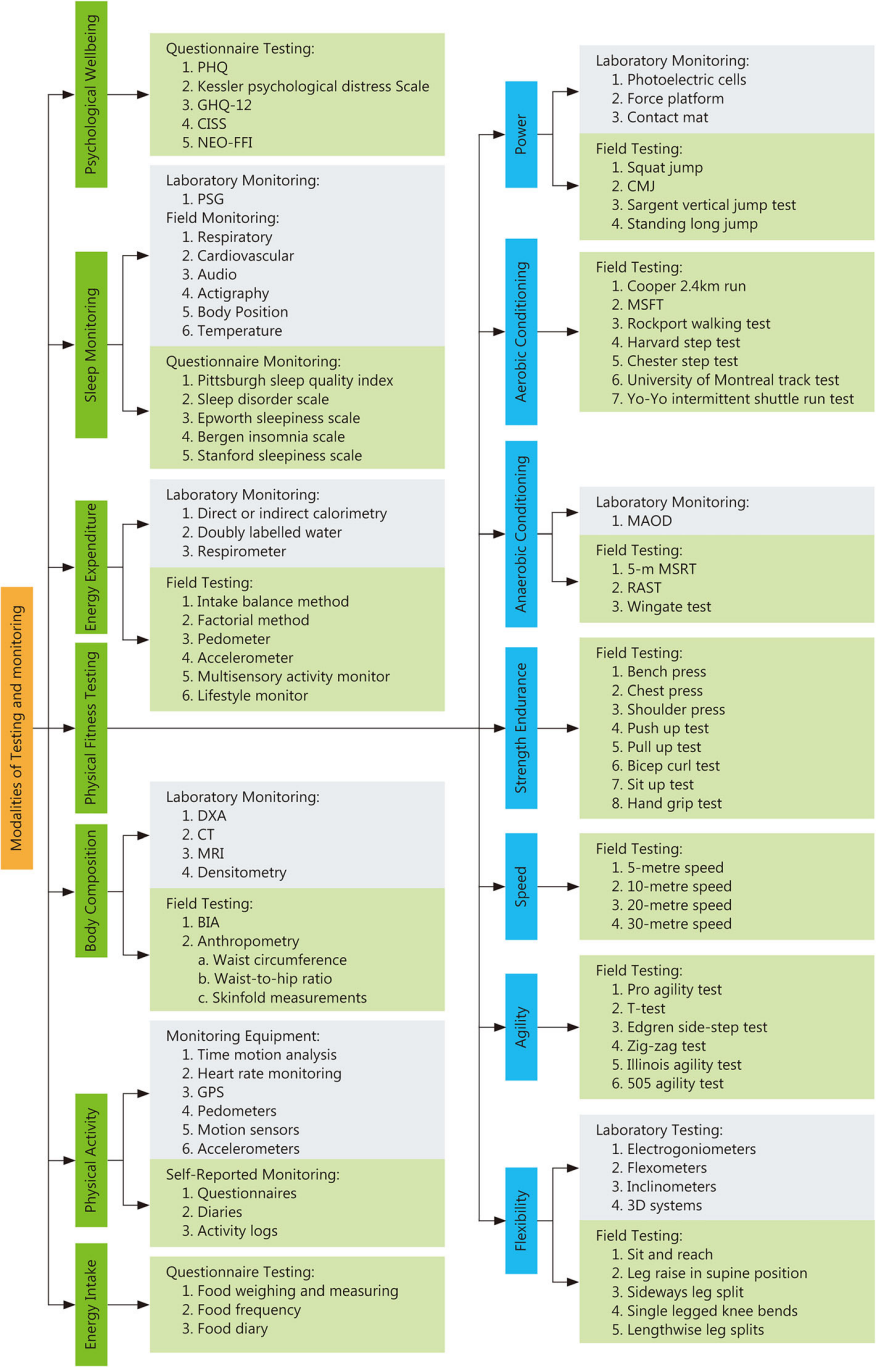
The methods of monitoring and assessing the physical and psychological wellbeing mentioned in this review. PHQ. Primary care evaluation of mental disorders patient health questionnaire; GHQ-12. General health questionnaire 12 item version; CISS. Coping inventory for stressful situations; NEO-FFI. NEO five factor inventory; PSG. Polysomnography; DXA. Dual energy x-ray absorptiometry; CT. Computed tomography; MRI. Magnetic resonance imaging; BIA. Bioelectrical impedance; GPS. Global positioning satellite system; CMJ. Counter movement jump; MSFT. Multi-stage fitness test; 5-m MSRT. 5-meter multiple shuttle run test; RAST. Running based anaerobic sprint test

Figure 1 outlines the methods of monitoring and assessing the physical and psychological wellbeing discussed throughout this review. Further research should also analyze how an individual’s physical fitness changes as a result of spending time at sea. If each component is considered separately, then it may be possible to establish trends according to factors such as age, gender or occupation, which in turn will allow for individualized testing and training programs within naval service cohorts and the tailoring of these programs to occupational relevance. When creating these fitness programs researchers also need to analyze the on-board environment in which an individual may spend quite a lot for time. A number of barriers to exercise may exist that will influence physical fitness levels and impact occupational effectiveness.

Most of the research conducted into the psychological wellbeing of naval service personnel looks at how individuals have been psychologically affected by particular stressors. Military organizations attempt to prepare individuals for the physical stressors that may be experienced and as such research should also look at how to prepare individuals psychologically for the stressors that may be experienced, as psychological wellbeing plays a significant role in the overall wellbeing of an individual. Additionally, the ability to cope in stressful situations or trends in personality traits should be analyzed to ascertain whether certain individuals may be more suited to specific occupations within the naval service.

The overall aim for each naval organization should be to holistically examine and optimize the methods required for screening and assessing the fitness of new recruits. Moreover, careful design of on board facilities and health maintenance programs for personnel on board should be encouraged to promote an integrated culture of health and fitness within navy cohorts.

## Conclusion

The aim of this paper was to analyze the research that exists on naval service personnel and to give a clearer understanding of the lifestyle and health factors that impact life at sea. From the data outlined in this article, it is clear to see that although there are many issues that have an effect on naval service personnel, none of these are completely unrelated. Fatigue could lead to a lack of energy to perform exercise on-board, a lack of exercise may lead to obesity as a result of over eating or a lack of control over diet, and obesity can lead to a lack of ability to perform occupational tasks that could affect mental wellbeing or stress, in turn leading to fatigue. It is therefore essential that when the wellbeing of naval service personnel is being assessed or prevention/intervention programs are being introduced, that it is not the only element that is focused on but is considered holistically. This type of a review is important not only for military cohorts but also for civilian occupations, where the wellbeing of employees is highly dependent on factors external to the individual’s control. These types of civilian occupations would not only include general seafarers but also fire fighters, police officers or miners, whose physical and mental occupational demands can be high and for whom issues such as fatigue can have a huge impact on occupational capabilities.

## Abbreviations

BIA, Bioelectrical impedance; BMI, Body mass index; CISS, Coping inventory for stressful situations; CMJ, Counter movement jumps; CT, Computed tomography; DXA, Dual energy X-ray absorptiometry; GHQ-12, General health questionnaire 12 item version; GPS, Global positioning satellite system; MAOD, Maximum accumulated oxygen deficit; MRI, Magnetic resonance imaging; MSFT, Multi-stage fitness test; NEO-FFI, NEO five factor inventory; NSWW, Navy standard working week; PHQ, Primary care evaluation of mental disorders patient health questionnaire; PLC-C, Post-traumatic stress disorder checklist-civilian version; PSG, Polysomnography; PTSD, Post traumatic stress disorder; RAST, Running based anaerobic sprint test; SWSD, Shift work sleep disorder; WHO World Health Organization.
